# Gene expression changes in male and female rhesus macaque 60 days after irradiation

**DOI:** 10.1371/journal.pone.0254344

**Published:** 2021-07-21

**Authors:** Matthäus Majewski, Patrick Ostheim, Zoya Gluzman-Poltorak, Vladimir Vainstein, Lena Basile, Simone Schüle, Michael Haimerl, Christian Stroszczynski, Matthias Port, Michael Abend

**Affiliations:** 1 Bundeswehr Institute of Radiobiology, Munich, Germany; 2 Department of Urology, Bundeswehr Hospital Ulm, Ulm, Germany; 3 Neumedicines Inc, Pasadena, CA, United States of America; 4 Applied Stem Cell Therapeutics, Milpitas, CA, United States of America; 5 Hadassah Medical Center, Jerusalem, Israel; 6 Department of Radiology, University Hospital Regensburg, Regensburg, Germany; Helmholtz Zentrum München, GERMANY

## Abstract

**Purpose:**

Transcriptome changes can be expected in survivors after lethal irradiation. We aimed to characterize these in males and females and after different cytokine treatments 60 days after irradiation.

**Material and methods:**

Male and female rhesus macaques (n = 142) received a whole-body exposure with 700 cGy, from which 60 animals survived. Peripheral whole blood was drawn pre-exposure and before sacrificing the surviving animals after 60 days.

**Results:**

We evaluated gene expression in a three-phase study design. Phase I was a whole-genome screening (NGS) for mRNAs using five pre- and post-exposure RNA samples from both sexes (n = 20). Differential gene expression (DGE) was calculated between samples of survivors and pre-exposure samples (reference), separately for males and females. 1,243 up- and down-regulated genes were identified with 30–50% more deregulated genes in females. 37 candidate mRNAs were chosen for qRT-PCR validation in phase II using the remaining samples (n = 117). Altogether 17 genes showed (borderline) significant (t-test) DGE in groups of untreated or treated animals. Nine genes (*CD248*, *EDAR*, *FAM19A5*, *GAL3ST4*, *GCNT4*, *HBG2/1*, *LRRN1*, *NOG*, *SYT14*) remained with significant changes and were detected in at least 50% of samples per group. Panther analysis revealed an overlap between both sexes, related to the WNT signaling pathway, cell adhesion and immunological functions. For phase III, we validated the nine genes with candidate genes (n = 32) from an earlier conducted study on male baboons. Altogether 14 out of 41 genes showed a concordantly DGE across both species in a bilateral comparison.

**Conclusions:**

Sixty days after radiation exposure, we identified (1) sex and cytokine treatment independent transcriptional changes, (2) females with almost twice as much deregulated genes appeared more radio-responsive than males, (3) Panther analysis revealed an association with immunological processes and WNT pathway for both sexes.

## Introduction

Gene expression changes caused by high dose irradiation have been investigated intensively over the last decades [1]. Radiation-induced long-term effects are known [2] and for example, stable chromosomal aberrations changes after high dose irradiation are used for retrospective dosimetry [3]. Effects of high dose irradiation include cancer as well as chronic non-cancer diseases [4–6] affecting e.g. the cardiovascular [7] and central nervous system [8, 9]. As almost two-third of all cancer patients undergo partial or whole body radiotherapy [10], the overall number of people with radiation-associated long-term effects is high making the topic pivotal [11]. Delayed effects of acute radiation exposure (DEARE) are other known health effects occurring after high radiation exposures. They are associated with degenerative and inflammatory conditions affecting multiple organs [12] and induce cerebrovascular injury [13] or lung injury [14]. Additionally, high dose irradiation with potentially lethal dose levels might occur [15] during large-scale radiological events like nuclear power plant disasters, the use of radiological weapons by terrorists, or during military conflicts. DEARE or long term effects have to be expected in survivors of these scenarios [6, 16].

In irradiated healthy individuals so far little is known about long-term changes in the transcriptome/post-transcriptome [17–19]. Recently, we identified persistent gene expression changes in the peripheral blood of sub-lethally irradiated baboons up to about three months after exposure and discussed their impact on long-term health effects [19]. A persistent alteration of the immune response 30 days after high dose partial body irradiation was postulated by another group [18], but overall, the molecular mechanisms and the entanglement with known long-term effects are not well characterized [1].

We measured gene expression in pre- and post-exposure (60 days) peripheral blood samples of irradiated 142 male and female rhesus macaques. Altogether 60 animals survived a severe and potentially lethal exposure to 700 cGy for 60 days. We hypothesized that high dose irradiation leads to sex-specific changes in the transcriptome 60 days after irradiation and wanted to examine whether previous results of our group performed in baboons could be validated in another species and at least at one time point. In collaboration with Neumedicines Inc., we assessed blood samples obtained from rhesus macaque that were involved in a study to determine the efficacy of IL-12 or/and G-CSF in the treatment of the hematopoietic acute radiation syndrome. The study was designed under the FDA Animal Rule. We performed a whole-genome screening and identified protein-coding mRNA genes in survivors separately for males and females. These mRNAs were then validated using the gold standard qRT-PCR methodology for gene expression analysis. Animals were either untreated (vehicle) or treated using recombinant human cytokines (IL-12), or IL-12 and G-CSF combined. As an additional step of validation, we did an inter-species comparison of candidate genes examined 60 days after irradiation with earlier generated results of a recent baboon study where we checked for long-term gene expression changes utilizing similar techniques and covering several months after irradiation.

## Materials and methods

### Animals and irradiation

Rhesus macaques were obtained from the Yongfu County Xingui Wild Animals Raising, China. At the beginning of the treatment, animal age ranged from 2 years, 5 months to 3 years, 10 months, and all animals weighed between 2.8 and 5.7 kg. Total body irradiation (TBI) comprised 700 cGy (60 cGy/min from a Theratron 1000 Co60 source [Best Theratronics; Ottawa, Ontario, Canada]). Details regarding animal characteristics as well as TBI procedure and other design details are described elsewhere [20]. Non-human primates (NHP) were randomized (separated by sex; stratified by weight) and either untreated (vehicle) or treated by subcutaneously administering either recombinant human IL12 (175 ng/kg), G-CSF (10 mg/kg), or a combination (for more details see [20]). Clinical signs were recorded twice daily and animals were euthanized at the occurrence of predefined criteria [20]. On day 60, all surviving animals (n = 60) were euthanized. Details of clinical assessments, supportive care, and euthanasia criteria are specified at [20]. Animals were housed individually in stainless steel cages equipped with an automatic watering system. Males and females were housed in separate rooms. The animal room environment was controlled to maintain a temperature of 21 ± 3°C; a relative humidity of 50 ± 20%; a light dark cycle of 12 hours light/12 hours dark, except during procedures; and 10–15 air changes per hour. Temperature and relative humidity were monitored continuously except during animal transportation and inside the radiation facility where only temperature was recorded every 5 minutes. A standard certified commercial chow (Harlan Teklad Certified Hi-Fiber Primate Diet #7195C) was provided to the animals twice daily except during designated procedures. Treats or fresh fruits/vegetables or juice (except orange or grapefruit juice) was provided as part of the animal enrichment program. If animals became appetiteless during the study, the standard diet was supplemented at the discretion of the Study Director or Clinical Veterinarian. Specifically, nutritional support (in the form of crushed cookies with banana, fruit and/or vegetable buffets, juice and/or Ensure®) was provided when body weight decreases by >15% of baseline or when an animal presented mouth lesion which hindered its food intake and was continued as long as the body weight remained >15% lower than baseline, and the animal was not eating. Municipal tap water (which has been exposed to ultraviolet light and purified by reverse osmosis) was provided to the animals ad libitum, except during procedures, via an automatic watering system or water bottles when considered necessary. Regarding symptomatic palliative care, buprenorphine was given for pain; bupivacaine (0.25%) was administered topically for the management of mouth ulcers; Pepto-Bismol was given for the management of diarrhea; topical hydrotherapy and/or iodine 1% was administered to wounds. Snacks or supplements (Rhesus Liquid Diet [BIO-SERV; Frenchtown, New Jersey], EnsureVR [Abbott Laboratories, Abbott Park, IL], vegetables, juices, or crushed cookies with banana) were given for anorexia. Animals in unrelievable pain or distress were euthanized based on the clinical judgment of the Clinical Veterinarians who was blinded to experimental treatments. Euthanasia endpoints were: Respiratory distress; Anorexia/Decreased appetite (Complete anorexia for 3 days with deteriorating conditions based on the clinical examination; animal not taking nutritive supplement offered orally); weight Loss, more than 20% over a 3-day period; decreased level of activity; acute, gross blood loss; status epilepticus; clinical appearance associated with abnormal vital signs. All procedures involving animals were conducted in compliance with the Good Laboratory Practice (GLP; 21 CFR Part 58) and performed at CiToxLAB North America (Montreal, Quebec, Canada) after approval by the Institutional Animal Care and Use Committee (IACUC) of CiToxLAB North America, Inc.

### RNA extraction and quality control

Whole blood samples (2.5 ml) were processed following the PAXgene Blood RNA System (BD Diagnostics, PreAnalytiX GmbH, Hombrechtikon, Switzerland). In brief, blood was drawn into a PAXgene Blood RNA tube at CiToxLAB. The tube was gently inverted (10 times), stored at room temperature overnight then at -20°. After all samples were collected, the PaxGene tubes were sent to Germany for further processing. After thawing, washing and centrifugation, cells in the supernatant were lysed (Proteinase K) followed by the addition of Lysis/Binding Solution taken from the mirVana Kit (Life Technologies, Darmstadt, Germany). With the mirVana kit, total RNA, including small RNA species, was isolated by combining a Phenol-Chloroform RNA precipitation with further processing using a silica membrane. After several washing procedures, DNA residuals became digested on the membrane (RNAse free DNAse Set, Qiagen, Hilden, Germany). RNA was eluted in a collection tube and frozen at -20°C. Quality and quantity of isolated total RNA were measured spectrophotometrically (NanoDrop, PeqLab Biotechnology, Erlangen, Germany). RNA integrity was assessed by the 2100 Agilent Bioanalyser (Life Science Group, Penzberg, Germany) and DNA contamination was controlled by conventional PCR using an actin primer. We used only RNA specimens with a ratio of A_260_/A_280_ ≥ 2.0 (Nanodrop) and RNA integrity number (RIN) ≥ 7.2 for RNA seq NGS (IMGM Laboratories, Martinsried, Germany) or RIN ≥ 7.3 for qRT-PCR analyses or no signs of degradation in agarose gel electrophoresis.

### Phase I screening

**Fig 1:**
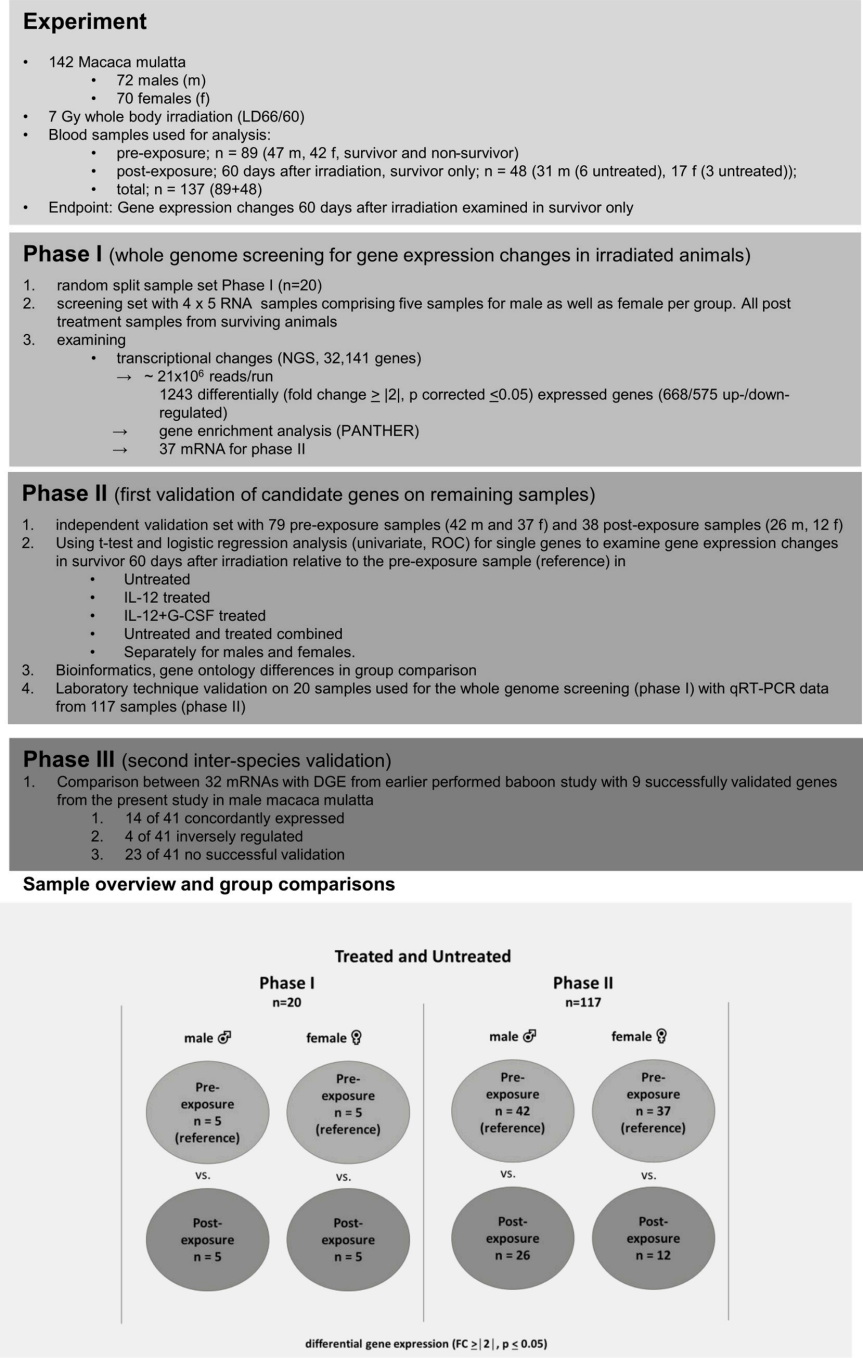
Flow diagram of included samples, three-phase study design, gene expression results, bioinformatics and inter-species validation between rhesus macaques and baboons. The overview shows the samples used in untreated (phase I) as well as untreated and treated rhesus macaques (phase II). Comparisons are shown separately for males and females in the lower panel for phase I and phase II.

*Whole-genome screening* for differentially expressed genes (protein-coding mRNAs) was performed on 20 RNA samples (four groups comprised pre- and post-exposure samples originating from five male and five female rhesus macaques, n = 4x5) among the untreated arm (Fig 1).

RNA Seq library preparation was based on the TruSeq® Stranded mRNA HT Library Prep Kit (Illumina, San Diego, CA) following the manufacturer’s recommendations (Illumina Inc. TruSeq® Stranded mRNA Sample Preparation Guide—Catalog # RS-122-9004DOC—Part # 15031047 Rev. E. 2013). In short, RNA was fragmented followed by cDNA synthesis, 3’ adenylation and adaptor ligation comprising indices for multiplex sequencing. After library quantification using the Qubit® dsDNA HS Assay Kit (Thermo Scientific, Waltham, MA) and assessment of length distribution using an Agilent Bioanalyzer system (Agilent 2100 Bioanalyzer, Agilent, Santa Clara, CA) equimolar pooling into a final sequencing library was performed. Before sequencing adapter dimers were removed using AMPure® XP Beads (Beckman Coulter, Brea, CA) followed by additional quality control using library quantification and electrophoresis as described above. Subsequently, single-end sequencing (1x75bp) was performed on the Illumina NextSeq 500 sequencing platform (Illumina, San Diego, CA).

For in-depth analysis of differential gene expression, we used the CLC Genomics Workbench (CLC Bio-Qiagen, Aarhus, Denmark; version 9.0.1). After the import of read data into the analysis tool, sequence reads were mapped against the *Macaca mulatta* genome (Mm1_8.0.1, NCBI). The incorporated edgeR Bioconductor package was used for the identification of DGEs [21]. In this study, only those gene transcripts that had a call “present” in at least 60% of RNA specimens were included in the analysis of gene expression and only genes with FDR corrected p-values ≤ 0.05 and with fold changes >2/<-2 among compared groups were considered to represent a candidate gene for validation in phase II. We considered multiple comparisons in the bioinformatic approach and in the validation part (phase II) of our study where the number of hypotheses tested in parallel was reduced from about 32,141 transcripts (phase I) to 37 mRNAs (see below).

### Phase II: Validation of phase I candidate genes via qRT-PCR

We independently validated the mRNA candidate genes from phase I (screening with NGS) using the remaining RNA samples (n = 117, Fig 1) and employing qRT-PCR, the gold standard in quantitative gene expression analysis. These samples comprised 79 pre-exposure samples taken from survivor and non-survivor as well as 38 post-exposure samples taken from survivor only (Fig 1). This strategy increased the sample size available for statistical analysis. We used a custom low density array (LDA, high throughput qRT-PCR platform) for quantification of our 37 candidate genes employing TaqMan chemistry (S1 Table). A 1 μg RNA aliquot of each RNA sample was reverse transcribed using a two-step PCR protocol (High Capacity Kit). 400 μl cDNA (1 μg RNA equivalent) was mixed with 400 μl 2 x RT-PCR master mix and pipetted into the fill ports of the LDA. Cards were centrifuged twice (1,200 rpm, 1 min, Multifuge3S-R, Heraeus, Germany), sealed, and transferred into the 7900 qRT-PCR instrument. The qRT-PCR was processed following the qRT-PCR protocol for 384-well LDA format.

For the LDA Ct-values (threshold cycles) we used the median mRNA expression on each LDA for normalization purposes, because this proved to be the more robust and the slightly more precise method compared to a normalization approach using a housekeeping mRNA species provided on the LDA.

All technical procedures for qRT-PCR were performed in accordance with standard operating procedures implemented in our laboratory in 2008 when the Bundeswehr Institute of Radiobiology became certified according to DIN EN ISO 9001/2008. All chemicals for qRT-PCR using TaqMan chemistry were provided by Life Technologies, Darmstadt, Germany.

### Phase III: Inter-species validation

The results from phase II were compared to whole-genome screening results from our earlier conducted male baboon study [19] (S2 Table). Altogether 32 candidate genes from the baboon screening data (Microarray) were compared to rhesus macaques data (NGS). Vice versa, nine candidate genes, that were positively validated in rhesus macaques (NGS) from the current study were examined in an already generated baboon whole genome (microarray) screening data file [19]. Only male individuals were included, as the baboon study did not involve females. The pre-treatment samples served as reference. Inter-species validation was defined as a fold change (FC) ≥ |1.5| in the comparison group. This less robust FC was chosen to adjust for methodological differences inherent to both studies and to avoid false negative results.

### Bioinformatics

For differentially expressed genes of Phase I we performed gene set enrichment analysis using PANTHER pathway software (http://www.pantherdb.org/, version 15, released 2020-02-14 [22]. PANTHER groups genes with similar biological functions based on their annotation (reference list was the current homo sapiens GO database). The p-values were corrected for multiple testing employing the False Discovery Rate algorithm (Table 1).

**Table 1:** Gene ontology (Panther) classification of differentially expressed genes.

**up-regulated genes**				
**Classification**	**male**		**female**	
	fold enrichment	p-value*	fold enrichment	p-value*
**GO biological process complete**				
anatomical structure regression	36.13	4.14E-02		
gland morphogenesis	9.67	5.57E-03		
positive regulation of T cell activation	7.08	1.83E-03		
positive regulation of leukocyte cell-cell adhesion	6.48	3.00E-03		
regulation of leukocyte cell-cell adhesion	4.99	1.61E-02		
regulation of cell-cell adhesion	4.38	1.06E-02		
regulation of cell adhesion	3.33	1.28E-02		
positive regulation of cell-cell adhesion	6.7	5.11E-04		
positive regulation of cell adhesion	4.44	5.10E-03		
positive regulation of lymphocyte activation	4.71	2.33E-02		
positive regulation of leukocyte activation	4.24	4.26E-02		
positive regulation of cell activation	4.13	4.91E-02		
regulation of T cell activation	5.36	4.69E-03		
T cell differentiation	7.02	1.06E-02		
T cell activation	6.2	4.07E-03		
immune system development	3.34	1.28E-0		
translation	4.2	5.58E-04		
peptide biosynthetic process	4.1	4.74E-04		
peptide metabolic process	3.56	1.18E-03		
cellular amide metabolic process	2.91	4.79E-03		
amide biosynthetic process	3.8	5.03E-04		
cell-cell signaling	2.97	2.52E-02		
T cell costimulation			18.99	4.63E-02
positive regulation of T cell activation			5.03	4.60E-02
positive regulation of cell-cell adhesion			4.84	1.51E-02
regulation of ossification			5.58	6.83E-03
**GO molecular function complete**				
coreceptor activity	18.73	4.09E-02		
structural constituent of ribosome	7.31	2.47E-06		
structural molecule activity	4.35	1.11E-05		
cytokine receptor activity	7.28	3.12E-02		
cytokine binding	6.61	2.30E-02		
**GO cellular component complete**				
**protein complex involved in cell adhesion**	13.6	5.71E-03	12.91	2.46E-03
cytosolic large ribosomal subunit	10.12	8.92E-05		
cytosolic ribosome	8.09	1.51E-05		
ribosome	5.9	3.17E-06		
large ribosomal subunit	6.27	2.67E-03		
ribosomal subunit	6.21	2.20E-05		
tight junction	7.44	1.91E-02		
external side of plasma membrane	5.55	1.65E-05		
cell surface	3.02	5.06E-03		
side of membrane	4.54	1.62E-05		
**plasma membrane signaling receptor complex**	5.35	1.61E-02	5.29	4.69E-03
receptor complex	3.7	7.13E-03		
external side of plasma membrane			3.57	1.39E-02
side of membrane			3.4	2.21E-03
**PANTHER Pathways**				
Gonadotropin-releasing hormone receptor pathway			3.36	6.01E-02
**Wnt signaling pathway**	3.69	2.48E-02	3.16	3.02E-02
**PANTHER GO-Slim Molecular Function**				
cytokine receptor activity	7.55	3.82E-02		
cytokine binding	6.93	4.41E-02		
**PANTHER GO-Slim Biological Process**				
positive regulation of cytosolic calcium ion concentration	7.17	1.12E-02		
regulation of cytosolic calcium ion concentration	6.02	2.23E-02		
**Wnt signaling pathway**	6.02	4.95E-02	6.12	1.04E-02
**cell-cell signaling by wnt**	5.96	4.38E-02	6.06	9.58E-03
**cell surface receptor signaling pathway involved in cell-cell signaling**	6.3	2.21E-02	6.23	3.92E-03
cell surface receptor signaling pathway	3.19	6.92E-04		
transmembrane receptor protein serine/threonine kinase signaling pathway			6.39	2.20E-02
enzyme linked receptor protein signaling pathway			4.1	1.45E-02
cellular response to growth factor stimulus			6.2	1.32E-02
response to growth factor			6.2	1.44E-02
negative regulation of signal transduction			4.47	1.59E-02
negative regulation of signaling			4.29	1.29E-02
**PANTHER GO-Slim Cellular Component**				
external side of plasma membrane	6.18	1.70E-03		
side of membrane	4.75	3.88E-03		
cell surface	3.91	2.28E-02		
leaflet of membrane bilayer	4.75	2.58E-03		
dendritic spine			11.11	6.26E-02
neuron spine			11.11	5.36E-02
**down-regulated genes**				
**Classification**	**male**				**female**		
fold enrichment	p-value*	fold enrichment	p-value*
**GO biological process complete**				
DNA-dependent DNA replication	10.86	5.95E-03		
DNA replication	11.21	1.53E-04		
cell division	9.07	4.87E-04		
chromosome segregation	6.45	1.36E-02		
mitotic cell cycle process	5.85	4.04E-04		
mitotic cell cycle	5.09	1.42E-03		
cell cycle	4.23	1.88E-04		
cell cycle process	4.7	7.27E-05		
cellular response to cytokine stimulus	4.05	2.05E-02		
immune response	3.26	2.98E-02		
response to molecule of fungal origin				
response to external biotic stimulus			5.59	8.06E-22
response to biotic stimulus			5.54	9.15E-22
pentose-phosphate shunt, non-oxidative branch			46.08	1.14E-02
pentose-phosphate shunt			34.13	3.29E-04
glucose 6-phosphate metabolic process			19.4	3.85E-04
NADPH regeneration			30.72	4.57E-04
NADP metabolic process			18.43	4.70E-04
type I interferon signaling pathway			36.86	2.65E-05
cellular response to type I interferon			39.1	2.01E-06
response to type I interferon			30.72	7.97E-07
innate immune response			6.94	2.61E-14
defense response to other organism			6.54	1.73E-19
defense response			5.43	3.96E-20
immune response			4.6	3.69E-15
immune system process			3.93	2.01E-20
cytokine-mediated signaling pathway			3.89	3.11E-03
interferon-gamma-mediated signaling pathway			36.86	1.64E-02
cytidine to uridine editing			30.72	2.17E-02
response to peptidoglycan			26.33	2.93E-02
regulation of ribonuclease activity			26.33	2.92E-02
detection of other organism			23.63	9.83E-04
detection of external biotic stimulus			21.94	1.26E-03
positive regulation of cytokine production involved in inflammatory response			21.94	1.24E-03
regulation of cytokine production involved in inflammatory response			11.89	2.82E-03
negative regulation of viral genome replication			21.59	4.67E-10
regulation of viral genome replication			14.01	3.33E-08
negative regulation of viral life cycle			16.3	7.79E-09
response to interferon-alpha			20.48	4.65E-02
cytoplasmic pattern recognition receptor signaling pathway			20.48	4.63E-02
negative regulation of interleukin-10 production			18.9	1.12E-02
defense response to protozoan			18.9	4.10E-16
defense response to virus			17.55	4.10E-16
positive regulation of interleukin-6 production			14.18	1.55E-05
positive regulation of interleukin-8 production			12.93	2.91E-02
positive regulation of cytokine biosynthetic process			12.8	8.02E-03
regulation of toll-like receptor signaling pathway			12.71	2.11E-03
lipopolysaccharide-mediated signaling pathway			11.81	1.08E-02
toll-like receptor signaling pathway			12.71	9.38E-04
activation of MAPKK activity			10.97	1.35E-02
positive regulation of tumor necrosis factor production			9.83	5.88E-04
regulation of erythrocyte differentiation			9.6	2.05E-02
positive regulation of chemokine production			9.03	2.52E-02
regulation of defense response to virus			8.19	1.30E-02
granulocyte chemotaxis			7.06	2.05E-02
positive regulation of NF-kappaB transcription factor activity				
**GO molecular function complete**				
single-stranded DNA helicase activity	28.58	4.55E-02		
DNA helicase activity	12.58	1.87E-02		
DNA-dependent ATPase activity	9.31	1.83E-02		
ATPase activity	4.75	5.42E-03		
immune receptor activity	8.51	2.54E-02		
cytokine binding	7.85	3.11E-02		
microtubule binding	6.03	9.52E-03		
2’-5’-oligoadenylate synthetase activity			46.08	2.81E-02
pattern recognition receptor activity			20.48	2.48E-02
double-stranded RNA binding			8.11	1.55E-02
**GO cellular component complete**				
replication fork protection complex	44.46	1.83E-02		
Midbody	7.69	2.74E-02		
Spindle	5.83	8.50E-03		
external side of plasma membrane	5.49	8.90E-03		
**PANTHER Pathways**				
Pentose phosphate pathway			24.57	9.35E-03
Toll receptor signaling pathway			5.76	4.89E-02
**PANTHER GO-Slim Molecular Function**				
cytokine receptor activity	9.95	1.06E-01		
cytokine binding	9.14	5.16E-02		
**PANTHER GO-Slim Biological Process**				
cell motility	4.61	4.35E-02		
localization of cell	4.61	5.80E-02		
organelle fission	4.45	5.31E-02		
cell cycle	3.81	1.03E-01		
defense response to virus			24.57	6.97E-10
negative regulation of cell-cell adhesion			14.46	3.79E-02
regulation of T cell proliferation			12.93	5.16E-02
T cell proliferation			12.93	4.89E-02
regulation of multi-organism process			9.64	6.40E-04
RNA phosphodiester bond hydrolysis			8.3	4.80E-02
**PANTHER Protein Class**				
Apolipoprotein	21.34	1.18E-02		
basic leucine zipper transcription factor			13.03	5.87E-04

Using the differentially expressed genes (DEG) from irradiated male and female rhesus macaque, a classification of overrepresented and underrepresented genes coding, e.g., biological processes or protein classes, was conducted using the bioinformatic tool PANTHER (http://www.pantherdb.org; version 15) which comprises Gene Ontology (GO) annotations directly imported from the GO database. Based on the comparison of observed vs expected numbers of upregulated or downregulated genes (reference database was Macaca mulatta) a fold enrichment in the number of genes annotated to this process and a corresponding p value (False Discovery Rate (FDR) corrected) was calculated. Numbers highlighted in grey refer to processes that were similar between male and female rhesus macaque.

### Statistical analysis

Quantitative gene expression data of the 37 candidate genes from phase II were examined using descriptive statistics (n, mean, standard deviation) and groups were compared employing the t-test. We assessed the assumptions of normality and equal variance and if required utilized either the pooled (equal variance) or the Satterthwaite variant of the t-test (unequal variance). Unconditional logistic regression analysis was performed for each of the independent variables (genes) of interest separately (univariate) on the binary outcome variable where the probability modeled was gene expression changes measured in the 60-day post-exposure sample relative to the pre-exposure sample used as the reference. Using logistic regression, we determined the area under a receiver-operator characteristic (ROC) curve providing a reasonable indication of overall diagnostic accuracy. ROC areas of 1.0 indicate complete agreement between the predictive model and the binary group comparison and thus a clear distinction between a pre- and a post-exposure sample. All calculations were performed using SAS (release 9.4, Cary NC, USA).

## Results

### Material available for the three-phase study design

For this study, Neumedicines Inc entrusted peripheral blood samples from 142 rhesus macaque comprising 72 samples from males and 70 from females (Fig 1, upper panel). Based on RNA quality and quantity (availability) criteria altogether 137 RNA samples from 113 rhesus macaque were eligible for this analysis (Fig 1).

During the *screening approach (phase I)*, we assessed 20 whole-genome mRNA sequencing (RNA seq) for 20 blood samples, randomly selected from the group of untreated NHP. Samples were collected before (pre-exposure) and 60 days after irradiation (post-exposure) from surviving animals (Fig 1, overview upper panel and sample overview lower panel). Five RNA samples were utilized for each, the pre- and post-exposure groups as well as males and females, summing up to 20 NGS examinations (n = 4x5). All samples originated from different animals, increasing inter-individual variance and decreasing false positive results occurring by chance.

For the *validation* of mRNAs, we used the remaining 117 blood samples comprising another 55 and 62 RNA samples from untreated and treated NHP, respectively (Fig 1, sample overview, phase II panel and lower panel). In phase II, we performed the validation separately for males and females, in groups of untreated (vehicle), treated (IL-12 and IL-12 + G-CSF combined) and merged untreated and treated NHP, thereby increasing the sample size examined.

### Phase I: RNA isolation and screening results

From 2.5 ml whole blood we isolated 10.6 μg (stdev 5.1 μg) total RNA on average before irradiation. RNA integrity (RIN) with a mean value of 8.0 (stdev 2.3) suggested high-quality RNA sufficient for running both methods.

The quality parameter for a successful sequencing run expressed as the percentage of Q30 bases (translates into a 99.9% accuracy) is supposed to be > 80% and was on average 90.6% in our study. The average number of PF reads (passed filter reads) was 21.6 x 10^6^ (stdev 3.8 x 10^6^) and ranged between 17.3–32.5 x 10^6^ reads/run. In total, 87–91% of the reads mapped to known gene regions (exons and introns).

**Fig 2:**
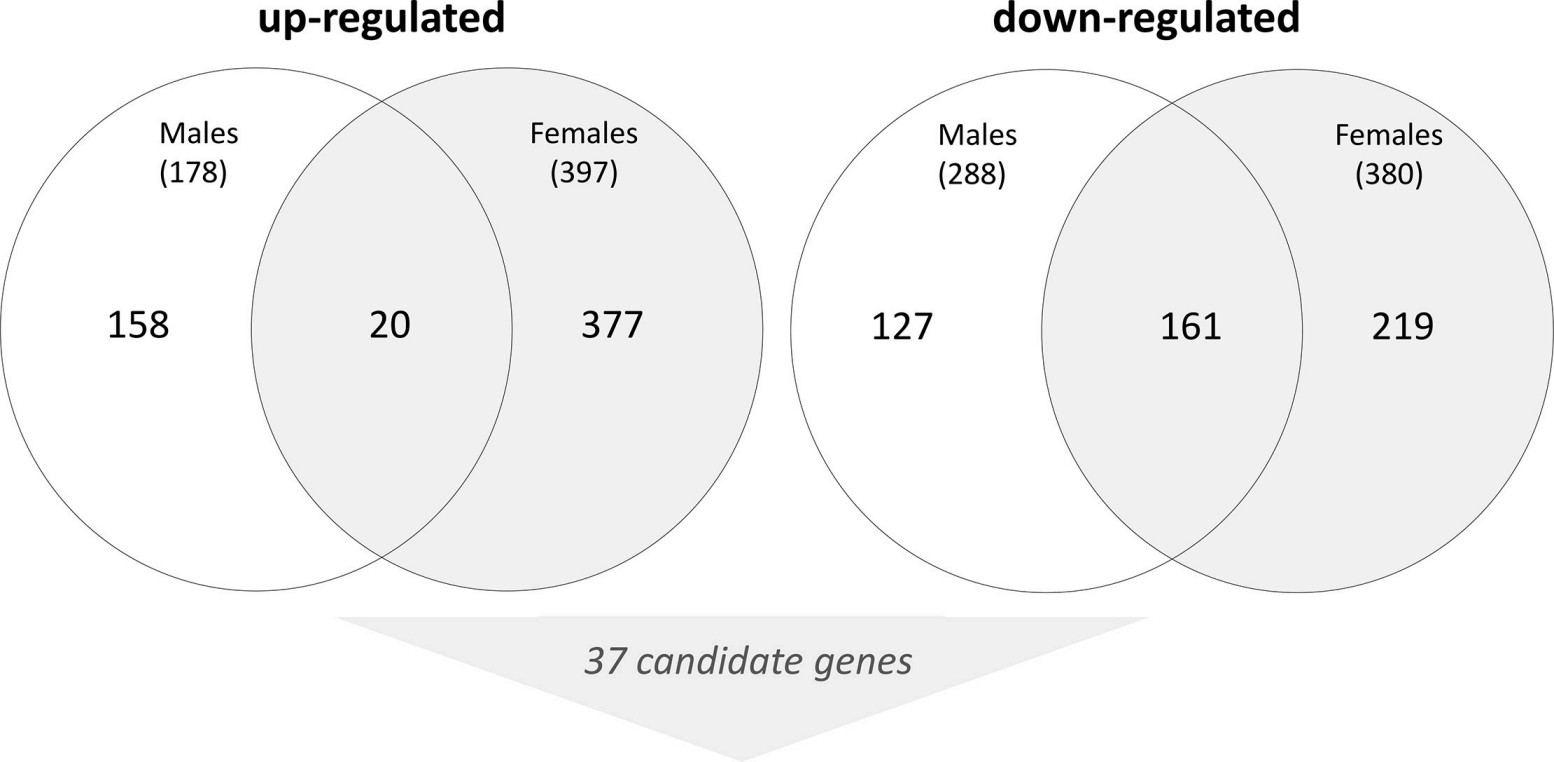
Venn diagram of up- and down-regulated genes that were differentially expressed by pre- and postexposure samples in male and female rhesus macaques. An overlapping number of genes being similarly up- and down-regulated in both sexes are presented by the numbers in the overlapping circles. The number of genes differentially expressed in males or females only is shown outside the overlapping circle. Numbers in parenthesis represent the total number of differentially expressed genes in males and females. Based on the fold-change, the p-value and the preference to genes differentially expressed in both sexes, 37 candidate genes were selected and put forward in phase II for validation purposes.

Based on our filter (Fold change ≥ |2|, p<0.05) we identified 1,243 DEG (668 up-regulated, 575 down-regulated, Fig 1) with 161 and 20 of them being similarly up-regulated and down-regulated in both sexes (Fig 2). Bioinformatic analysis regarding the significant enrichment of genes to certain gene ontology (GO) classifications revealed a small overlap between both sexes, namely related to the WNT signaling pathway (males: fold enrichment, 6.02, p-value 0.049; females: fold enrichment 6.12, p-value 0.01) and cell adhesion (males: fold enrichment, 13.6, p-value 0.006; females: fold enrichment 12.91, p-value 0.0025; Table 1). Genes coding for immunological functions (e.g. T-cell costimulation or leukocyte cell-cell stimulation) were differently enriched in both sexes. The number of deregulated genes was about 30–50% higher in females relative to males and about 2-time more genes appeared up-regulated relative to the down-regulated genes in both sexes (Fig 2). Using the fold-difference and the p-value, we selected 37 most promising candidate mRNAs for validation at phase II employing qRT-PCR.

### Phase II: Independent methodological validation of NGS using qRT-PCR

In a first step, where we examined for genes expressed in 117 RNA samples, we identified 20 out of 37 candidate genes eligible for analysis (S1 Fig). Thirteen of these mRNAs independently examined in 117 samples using qRT-PCR appeared up-regulated (*HBG2/1*) or down-regulated similarly to the other 20 RNA samples used to employ the NGS whole-genome screening (S1 Fig). False-positive results (control values detected with qRT-PCR, while NGS data indicated an up- or down-regulation) were identified in six mRNAs and an inverse regulation with both methods could be detected in one case.

**Fig 3:**
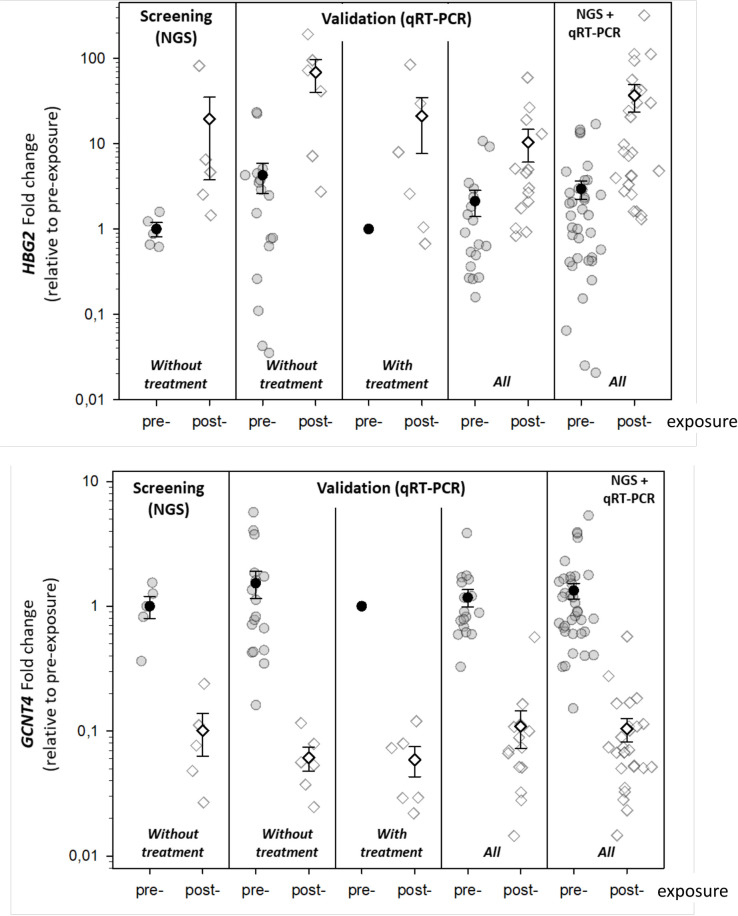
The jitter plots represent fold-changes in gene expression calculated between post-exposure samples relative to the pre-exposure samples, with the latter arbitrarily set to the value one. Single measurements (symbols with grey edging) as well as mean values (black edged symbols, error bars do show the standard error) of two representative genes are shown. Gene expression data from pre- and post-exposure samples used for screening (left side) or validation (right side) are shown in untreated, treated (IL12, IL12+G-CSF) and both groups combined “All”. The last plots to the right reveal gene expression measurements of all samples (screening as well as validation). Fold changes for the no treatment group were calculated based on untreated pre-exposure samples, for “all” they were calculated based on pre-exposure samples from the no treatment, IL12 and IL12+G-CSF group to ensure comparability.

From these 13 validated candidate mRNA species appeared expressed and showed significant or borderline significant DGE in post-exposure relative to pre-exposure samples in separate analysis considering sexes and treatment groups. In a second analysis step, we considered significant changes and a minimal number of 50% expressed samples per group. Based on that, nine most promising validated genes were identified, namely *CD248*, *EDAR*, *FAM19A5*, *GAL3ST4*, *GCNT4*, *HBG2*, *LRRN1*, *NOG* and *SYT14* (Table 2). All nine genes revealed the same direction in DGE in the post-exposure samples relative to the pre-exposure sample irrespective of the examined group of untreated or cytokine treated NHP or males and females (Table 2). This is graphically visualized for *HBG2/1* (up-regulated) and *GCNT4* (down-regulated) using the NGS screening data and the corresponding qRT-PCR data which reveal the results separately for untreated, cytokine treated and merged untreated and treated groups (Fig 3). The last plot in Fig 3 combines NGS and all qRT-PCR data.

**Table 2:** Overview of nine genes showing significantly different DGE in post-exposure relative to pre-exposure samples.

		Males							Females						
treatment	mRNA species	n (pre-exposure)	n (60 days after irr.)	mean Ct-value (pre-exposure)	mean Ct-value (60 days after irr.)	Fold-change	p-value	ROC	n (pre-exposure)	n (60 days after irr.)	mean Ct-value (pre-exposure)	mean Ct-value (60 days after irr.)	Fold-change	p-value	ROC
without treatment	*CD248*	14	1	15.3	17.8	0.2		1.0	22	2	15.5	17.7	0.2	0.08	0.91
with treatment, IL-12		1	3	15.1	17.7	0.2		1.0	5	1	14.8	17.7	0.1		1.0
with treatment, IL-12+G-CSF		18	3	15.0	17.8	0.1	0.002	1.0	9	2	14.4	16.0	0.3	0.027	0.94
all		33	7	15.1	17.8	0.2	**<0.0001**	1.0	36	5	15.1	16.7	0.3	**0.003**	0.87
without treatment	*EDAR*	16	2	13.6	17.6	0.1	0.002	1.0	22	3	13.3	15.4	0.2	0.0002	0.99
with treatment, IL-12		1	5	12.5	16.5	0.1		1.0	5	3	13.6	17.5	0.1	0.0001	1.0
with treatment, IL-12+G-CSF		18	8	13.38	17.38	0.1	<0.0001	1.0	9	3	13.1	16.2	0.1	0.001	1.0
all		35	15	13.5	17.1	0.1	**<0.0001**	0.99	36	9	13.3	16.4	0.1	**<0.0001**	0.99
without treatment	*FAM19A5*	15	3	15.16	17.3	0.2	0.08	0.87	17	2	15.9	16.7	0.6	0.4	0.69
with treatment, IL-12		1	5	12.5	16.1	0.1		1.0	3	3	17.2	17.1	1.0	0.95	0.5
with treatment, IL-12+G-CSF		15	9	15.9	17.1	0.4	0.012	0.83	8	3	15.2	17.1	0.3	0.11	0.88
all		31	17	15.4	16.9	0.4	**0.0021**	0.78	28	8	15.8	17.0	0.4	**0.004**	0.75
without treatment	*GAL3ST4*	16	2	14.2	17.6	0.1	0.08	1.0	22	2	14.4	15.7	0.4	0.07	1.0
with treatment, IL-12		1	4	13.95	17.4	0.1		1.0	5	1	14.9	17.1	0.2		1.0
with treatment, IL-12+G-CSF		18	5	14.8	17.5	0.2	<0.0001	0.99	9	3	14.1	16.2	0.2	0.15	
all		35	11	14.5	17.5	0.1	**<0.0001**	0.99	36	6	14.4	16.2	0.3	**<0.0001**	0.92
without treatment	*GCNT4*	17	6	11.2	15.4	0.1	<0.0001	1.0	23	3	11.3	12.9	0.3	0.095	0.94
with treatment, IL-12		1	6	9.9	14.3	0.0		1.0	5	3	11.0	15.2	0.1	<0.0001	1.0
with treatment, IL-12+G-CSF		18	14	11.1	14.9	0.1	<0.0001	1.0	9	6	10.6	14.9	0.1	0.004	1.0
all		36	26	11.1	14.9	0.1	**<0.0001**		37	12	11.1	14.5	0.1	**<0.0001**	0.96
without treatment	*HBG2_HBG1*	18	6	10.18	5.17	32.2	0.002	0.91	23	3	8.5	10.6	0.2	0.1	0.87
with treatment, IL-12		1	6	7.5	5	5.7			5	3	9.8	4.8	32.0	0.035	1.0
with treatment, IL-12+G-CSF		18	14	8.75	6.6	4.4	0.0025	0.86	9	6	9.1	5.5	12.1	0.048	0.83
all		37	26	9.4	5.9	11.3	**<0.0001**	0.86	37	12	8.8	6.6	4.6	0.07	0.68
without treatment	*LRRN1*	21	5	12.24	15.52	0.1	0.0008	0.93	22	3	12.2	15.0	0.1	0.005	0.94
with treatment, IL-12		1	6	12	14.95	0.1		0.83	5	3	12.6	14.6	0.3	0.029	1.0
with treatment, IL-12+G-CSF		18	11	12.8	16.3	0.1	<0.0001	0.99	9	5	12.5	14.8	0.2	0.014	0.89
all		40	22	12.5	15.77	0.1	**<0.0001**	0.94	36	11	12.3	14.8	0.2	**<0.0001**	0.92
without treatment	*NOG*	20	6	11.64	15.96	0.1	<0.0001	1.0	23	3	11.6	13.3	0.3	0.049	0.96
with treatment, IL-12		1	6	10.8	15.5	0.0		1.0	5	3	11.5	16.1	0.0	0.0002	1.0
with treatment, IL-12+G-CSF		18	14	11.3	15.5	0.1	<0.0001	1.0	9	6	10.7	15.2	0.0	0.004	
all		39	26	11.4	15.4	0.1	**<0.0001**		37	12	11.3	15.0	0.1	**<0.0001**	0.96
without treatment	*SYT14*	15	1	16.04	17.69	0.3			22	2	15.5	16.8	0.4	0.15	0.86
with treatment, IL-12		1	2	15.5	16.4	0.5		1.0	5	1	15.8	18.0	0.2		1.0
with treatment, IL-12+G-CSF		18	4	15.9	17.2	0.4	0.008	0.9	9	3	15.4	16.2	0.6	0.2	0.72
all		34	7	16	17.1	0.5	0.011		36	6	15.5	16.7	0.4	0.016	0.8

Overview of nine genes that were significantly different in post-exposure relative to pre-exposure samples and expressed in ≥ 50% of samples per group. Data are presented separately for male (left part) and female rhesus macaques (right part) and ordered starting with untreated, treated (IL-12 and IL-12 + G-CSF), and untreated and treated groups combined (all). The number of measurements and the mean gene expression (threshold cycle, Ct) values are provided by pre- and post-exposure samples as well as the fold-change difference (derived from Ct-values applying the ΔΔ-Ct-approach). For instance, fold-changes of 0.1 or 10 are referring to a 10-fold down-regulation or up-regulation, respectively. A t-test was applied for this group comparison. P-values (chisquare statistic) of the category “all” are presented in bold when surviving Bonferroni correction for multiple comparison. ROC areas were derived from logistic regression analysis.

P-values of the nine most promising, validated mRNAs in Table 2 further decreased after merging treated and untreated NHP and all except one mRNA (*SYT14*) in males and females and another mRNA (HBG2/1) in females survived Bonferroni correction for multiple comparison (p<0.0056; 0.05/9, Table 2).

Eight genes were significantly down-regulated (3-10-fold) and *HBG2/1* (treatment group dependently) appeared about 10-32-fold up-regulated. Complete or almost complete separation of groups (ROC lying mostly between 0.8–1.0) was observed (Table 2).

### Phase III: Inter-species validation

**Fig 4:**
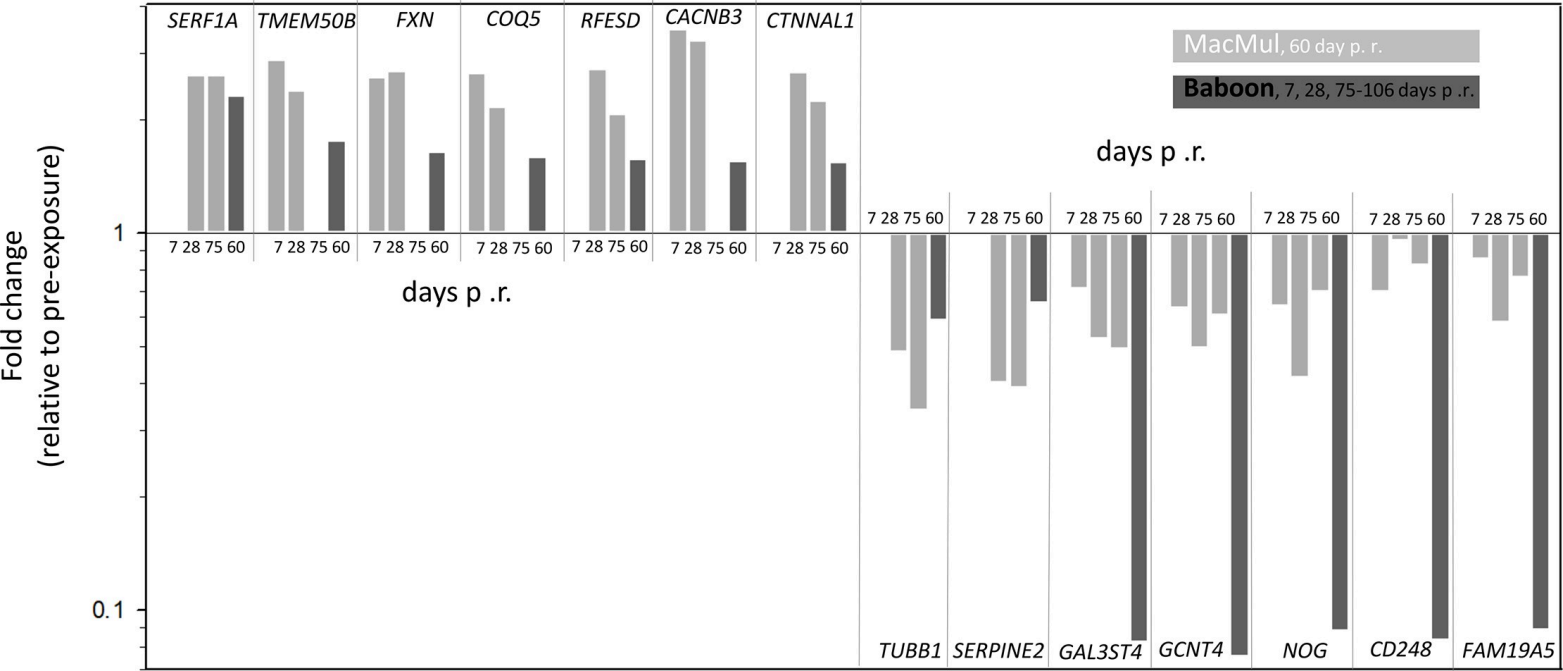
Bar graphs represent the fold changes of 14 successfully validated candidate genes originating from Baboon (light gray, 1–3 time points after irradiation) respectively Macaca mulatta (dark grey, 60 days after irradiation) from S2 Table. Fold changes are shown relative to pre-treatment samples on a logarithmic scale (fold change ≥ |1.5|). p. r., post irradiation.

The nine successfully validated candidate genes (Table 2) from male rhesus macaques were compared to the whole genome microarray screening database from an earlier performed baboon study in males [19]. Vice versa, the 32 validated candidate genes from male baboons were compared to the NGS screening results of the current study on rhesus macaques. Overall, 34% of the genes (14 of the 41 genes (nine originating from rhesus macaques and 32 originating from a previous baboon study) showed a concordant DGE (FC) ≥ |1.5|; S2 Table), whereby nine baboon candidate genes and five rhesus macaques candidate genes could be validated in the other species (Fig 4). No inter-species validation could be performed in 23 candidate genes from both species together. Four genes were inversely regulated and 23 could not be validated between the two species (Fig 4).

## Discussion

In developed countries about two-third of all cancer patients undergo radiotherapy and about half of them survive, so that a secondary tumor or chronic non-cancer late effects might develop [11, 23]. Individuals involved in the malevolent use of radiation sources or involved in an accident like a nuclear power plant disaster might be exposed to even higher, potentially lethal doses of radiation and survivor might develop delayed effects of acute radiation exposure (DEARE, [17–19]). Taking advantage of a non-human primate model with high genetic homology to humans, we examined for transcriptomic changes occurring in ARS survivor and compared our molecular results with published molecular changes known to be associated with later occurring health effects and DEARE.

In collaboration with Neumedicine Inc., we analyzed 137 RNA samples from 113 rhesus macaque originating from a clinical study to examine the effect of IL-12 and G-CSF on the survival of rhesus macaque after potentially lethal whole-body exposure. Male and female rhesus macaques were treated during this study, providing the opportunity to examine sex and treatment specific differences in gene-expression changes 60 days after exposure in comparison to the pre-exposure measurements.

Whole-genome bioinformatic analysis revealed a small overlap of sex-associated gene enrichment to certain gene ontology (GO) classifications. This overlap was mainly related to the WNT signaling pathway and immunological processes (Table 1). Previous studies already reported, that immunological alterations might play a role in post-radiation tumor development besides the direct carcinogenic effect of radiation [24]. Radiation exposure that led to long-term gene expression changes even weeks after exposure [25–27] or DEARE [17–19] were already described in previous studies.

Because our measurements were performed on peripheral whole blood, presumably our results either reflect altered hematopoietic stem cells or indicated a selection for less radiosensitive stem cells reconstituting the peripheral blood after radiation exposure. That would explain the rather high fold-changes observed in several of the mRNA species and their persistency in unirradiated cellular progeny. It can be speculated whether our WNT pathway results might relate to it, since the canonical WNT pathway is known to promote hematopoiesis via β-catenin [28] and reports indicate a radiation-induced high activity of the canonical WNT pathway associated with increased DNA damage in murine hematopoietic stem cells [29]. Moreover, for the gastrointestinal tract, two functional distinct pools of intestinal stem cells are proposed [30]. Actively cycling, radiation-sensitive, WNT pathway modulated stem cells are discriminated from slowly cycling, radiation-resistant, WNT pathway refractory and injury inducible stem cells. After radiation injury, a shift in the composition of these differently radiosensitive stem cells after irradiation is expected in the gastrointestinal tract [30]. A mechanism like that could be proposed for the hematopoetic system. This shift in the composition of hematopoetic stem cells would be reflected by strong fold-differences in gene expression as observed in our study 60 days after irradiation relative to the pre-exposure measurements.

From the 37 selected candidate genes, nine appeared significantly deregulated (Table 1). These changes at 60 days after exposure were found to be similar in males and females and independent of the treatment regimen. Some of the validated genes were described to be coding for proteins involved e.g. in chemokine/cytokine function (*FAM19A5*, *NOG*) and extracellular matrix function (*CD248*, *GCNT4*) (S3 Table). Others are known for their association with certain diseases (*EDAR*, *GAL3ST4*, *LRRN1*, *SYT14*). Corresponding to our findings, a de-regulation of *HBG2/HBG1* was observed in peripheral blood of radiotherapy patients within 16 hours after 2 Gy radiation. Its role as potential predictor for radiation toxicity was discussed [31].

For further validation, we compared previously reported gene expression results from irradiated male baboons [19, 32] with male rhesus macaque data from the current study. Methodological differences in both experiments (microarrays in baboons versus NGS in this study) necessitated adjustments of the fold change to a less strict cut off value ((FC) ≥ |1.5|) to avoid false negative results. We earlier showed that even when organisms with a striking gene sequence homology are compared, inter-species differences might occur (inverse regulation of *FDXR* gene in baboons and humans after radiations [33]), which underlines this additional validation step. Altogether 34% of deregulated genes could be successfully validated in both species (Fig 4) [19], which further supports the significance of our findings. Examinations in baboons covered a period of about three months. As a limitation of our rhesus macaque study, we could perform gene expression measurements at 60 days after irradiation only. However, one third of the identified genes showed similar gene expression changes over the whole three months period of time in both species. This led us speculate, that the 60 days after irradiation identified rhesus macaque gene expression changes might reflect long lasting changes with impact on DEARE and late health effects as discussed above.

Because of sex and cytokine treatment differences (Fig 1) a separate analysis was performed. Similarities in gene expression independently of sex and treatment regimens observed in the majority (71–95%) of examined samples indicate that cytokine treatment (IL-12 and G-CSF) and sex differences for these genes do not affect gene expression changes at least 60 days after irradiation (Table 1). To our knowledge that has not been examined before in healthy irradiated NHP.

A higher radiosensitivity in females was discussed in the literature, especially in the context of second malignancies [34–36]. In our study, the number of deregulated genes was about 30–50% higher in females relative to males indicating females being more radio-responsive on the transcriptome level compared to males.

NGS results could be successfully validated with qRT-PCR in 13 but not all expressed 20 mRNAs. This might be improved in future studies when identifying with NGS not the radiation-induced gene, but the specific radiation-responsive exon-region of a gene contributing primarily to the radiation-induced gene expression changes and using a complementary TaqMan assay for q-RT-PCR validation, specifically covering this exon region.

Some limitations of our study should be kept in mind. Only one time point after irradiation (60 days) was available in this experiment and it is unclear whether the observed gene expression changes persist over time. However, the study design did not allow for further examinations on other time points [20], but corresponding changes of validated genes detected in baboons suggest a persistency over a larger time frame. The strength of our study certainly is a large number of animals as well as having males and females included. The validation was performed with high robustness by changing both, methodology and samples during the first validation step and performing an inter-species comparison as a second validation step.

In summary, 60 days after radiation exposure we identified (1) cytokine treatment independent transcriptional changes, (2) females with almost twice as much deregulated genes appeared more radio-responsive than males and (3) Panther analysis revealed a strong association with immunological processes as well as the WNT pathway for both sexes and these processes are known to be associated with secondary malignancies and other inflammatory associated diseases, e.g. lung fibrosis or cardiovascular diseases.

## Supporting information

S1 Fig Methodological comparison of NGS and qRT-PCR data using 20 expressed genes. Mean fold-changes are presented for animals used for NGS (n = 20) and the remaining animals using qRT-PCR (n = 117). Genes where a successful validation failed are shown in the inserted graph.
(TIFF)

S1 Table Overview of 37 TaqMan assays used for validation of screening results (NGS). Gene numbers and fold-changes (first number represents male and second number female fold-changes) and sexes are presented.
(XLSX)

S2 Table Comparison between screening results of Baboon microarray screening results (middle column, three time points (7, 28 and 75–106 days after irradiation) and NGS screening results from Macaca mulatta (right column, 60 days after irradiation) with validated genes of the Baboon and Macaca mulatta experiment. Data is sorted by successful inter-species validation (14/41), no inter-species validation (23/41) and inverse inter-species validation (4/41). Successfully validated time points are shown in bold.
(XLSX)

S3 Table Annotation of nine most promising genes for discrimination of post- from pre-exposure samples. A summary of the currently known function is given. Results for a Pubmed search (http://www.pubmed.gov) for “gene name” + “radiation” are given.
(XLSX)
