# Supplementary figures and images for: Gene expression changes in male and female rhesus macaque 60 days after irradiation
Source: PLoS One. 2021 Jul 21;16(7):e0254344. doi: 10.1371/journal.pone.0254344 (PMC8294544; doi:10.1371/journal.pone.0254344)

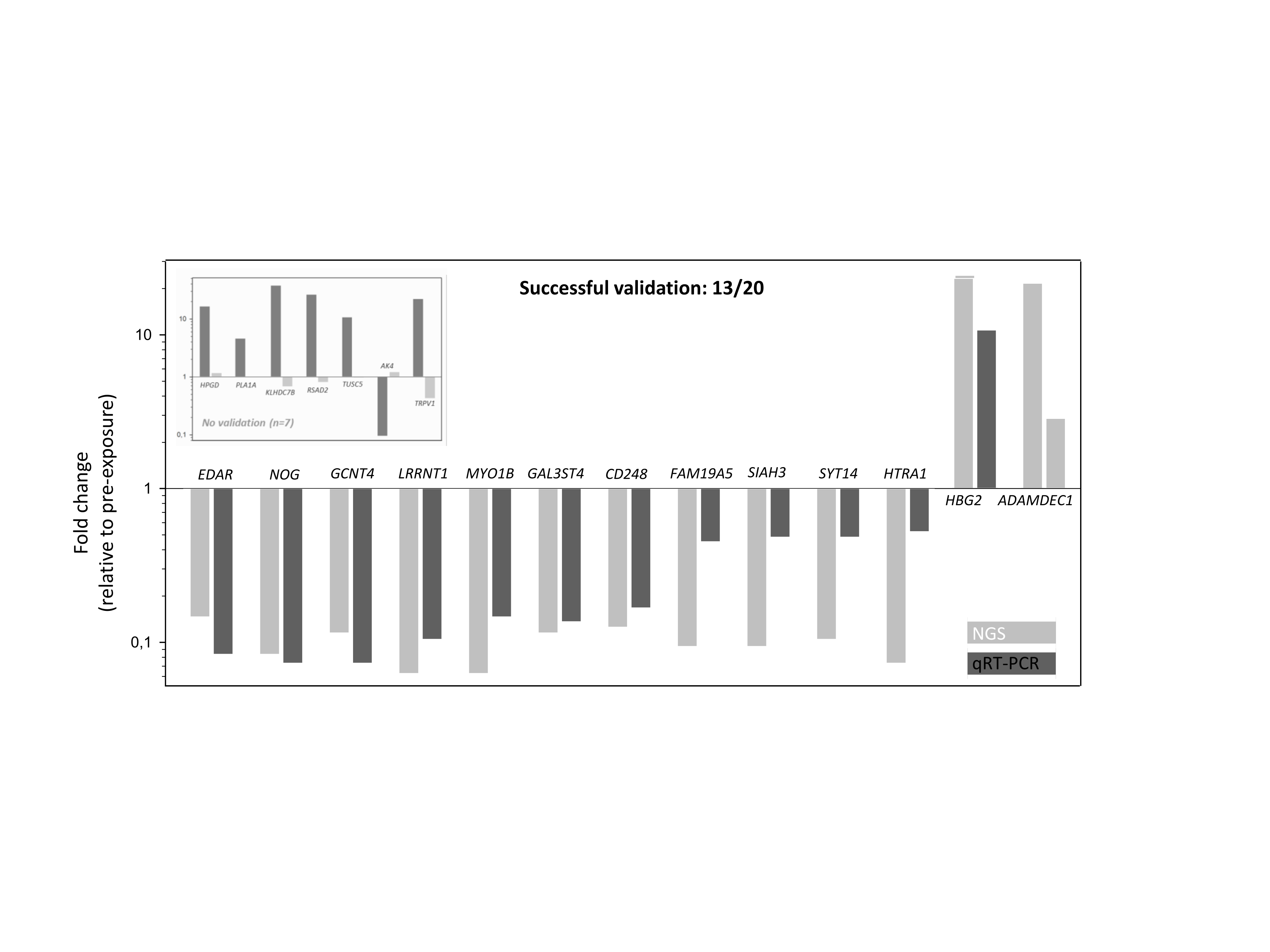

Supplement: S1 Fig — Mean fold-changes are presented for animals used for NGS (n = 20) and the remaining animals using qRT-PCR (n = 117). Genes where a successful validation failed are shown in the inserted graph. (TIFF) [file pone.0254344.s001.tiff]
